# Risk factors for periprocedural ischemic stroke following endovascular treatment of intracranial aneurysms

**DOI:** 10.1186/s41016-021-00255-z

**Published:** 2021-08-23

**Authors:** Yisen Zhang, Chao Wang, Zhongbin Tian, Wei Zhu, Wenqiang Li, Xinjian Yang, Jian Liu, Ying Zhang

**Affiliations:** Department of Interventional Neuroradiology, Beijing Neurosurgical Institute and Beijing Tian Tan Hospital, Capital Medical University, No.119, South 4th Ring West Road, Fengtai District, Beijing, 100070 China

**Keywords:** Periprocedural ischemic stroke, Endovascular treatment, Intracranial aneurysms, Aneurysm size, Treatment modality

## Abstract

**Background:**

The aim of this study was to comprehensively evaluate the risk factors of periprocedural ischemic stroke associated with endovascular treatment of intracranial aneurysms using a real-world database.

**Methods:**

From August 2016 to March 2017, 167 patients were enrolled. Univariate analysis and multivariate logistic regression analysis were used to examine the risk factors for periprocedural ischemic stroke.

**Results:**

Among the 167 cases, periprocedural ischemic stroke occurred in 20 cases (11.98%). After univariate analysis, the ischemic group had a higher proportion of large (≥ 10 mm) aneurysms than the control group (45.0% vs. 23.1%, *p* = 0.036). The incidence of periprocedural ischemic stroke was higher in cases treated by flow diverter (21.6%) or stent-assisted coiling (11.8%) than in cases treated by coiling only (2.7%), and the differences were statistically significant (*p* = 0.043). After multivariate logistic regression analysis, treatment modality was the independent risk factor for periprocedural ischemic stroke. Compared with the coiling-only procedure, flow diverter therapy was associated with a significantly higher rate of periprocedural ischemic stroke (OR 9.931; 95% CI 1.174–84.038; *p* = 0.035).

**Conclusions:**

Aneurysm size and treatment modality were associated with periprocedural ischemic stroke. Larger aneurysms were associated with increased risk of periprocedural ischemic stroke. Flow diverter therapy was associated with significantly more periprocedural ischemic stroke than the coiling procedure alone.

## Background

In recent decades, endovascular treatment has become a major modality in the treatment of intracranial aneurysms, because the safety and efficacy have been demonstrated in numerous large clinical studies [1–3]. With the endovascular device improvements and increasing clinical experience, intracranial aneurysms could be treated by a more rational modality according to patient or aneurysm characteristics, which increased the aneurysm occlusion rates [4]. For instance, the wide-neck aneurysms (≥ 4 mm or dome-neck ratio ≤ 2) were usually treated by intracranial stent [5]. But for large or complex aneurysms, flow diverter therapy is the preferred treatment [6, 7].

However, endovascular treatment of intracranial aneurysm has the risk of neurological complications. Periprocedural ischemic stroke is the most common neurologic complication following endovascular treatment of intracranial aneurysms and is a major source of neurological morbidity and mortality [8, 9]. A better understanding of the risk factors for periprocedural ischemic stroke is important and may prove beneficial in the decision-making process of intracranial aneurysm management. However, limited studies have comprehensively evaluated the risks of periprocedural ischemic stroke associated with endovascular treatment of intracranial aneurysm using a real-world database. Therefore, the aim of this study was to evaluate the incidence and risk factors of periprocedural ischemic stroke following endovascular treatment using a real-world database. The clinical, aneurysmal, and procedural characteristics associated with periprocedural ischemic stroke were analyzed in this study.

## Methods

### Patient selection

This study is a retrospective study and the data on patients with intracranial saccular aneurysms treated by endovascular treatment were collected form a real-world database (the HARET study) [10]. Briefly, patients with a single unruptured intracranial saccular aneurysms who underwent endovascular treatment in our institute between August 2016 and March 2017 were enrolled in the current study. The exclusion criteria included multiple aneurysms, ruptured aneurysm, intracranial aneurysm with previous treatment, treatment by parent vessel occlusion, treatment by a covered stent, presence of a brain arteriovenous malformation, and presence of a dissecting aneurysm. Ultimately, 167 eligible patients with 167 aneurysms were enrolled in this study. The study was approved by our institutional review board. All patients or their relatives provided written informed consent during hospitalization. This retrospective case analysis study examined the variables associated with periprocedural ischemic stroke. Clinical, aneurysmal, and procedural factors were recorded and analyzed. These factors were as follows: age, sex, history of alcohol intake and cigarette smoking, hypertension, hyperlipidemia, previous ischemic comorbidity, aneurysm size (maximum size), shape (irregular: blebs, nipples or multiple lobes), sidewall/bifurcation, location (anterior/posterior circulation), distal aneurysm (at or distal to the Circle of Willis), and treatment modalities (including coiling only, stent-assisted coiling or flow diverter treatment). The occurrence of periprocedural ischemic stroke within 30 days of the endovascular treatment was also recorded, and the 167 eligible patients were divided into two groups (ischemic group and control group). All patients and aneurysms with periprocedural ischemic complications were included in the ischemic group. A major ischemic event was defined as an event lasting for more than 7 days, and a minor ischemic event was defined as an event that resolved within 7 days with no clinical sequelae [11]. All major ischemic events were included in the neurologic morbidity and mortality rates.

### Endovascular procedures

For each endovascular procedure, the treatment strategy was discussed and chosen at the weekly peer-reviewed endovascular conference. Coiling alone was performed if the aneurysm neck was narrow (< 4 mm or dome-neck ratio > 2). For wide-neck aneurysms (≥ 4 mm or dome-neck ratio ≤ 2), the stent-assisted coiling technique was used. However, if the aneurysm was large or complex and therefore unsuitable for conventional endovascular treatment (coiling or stent-assisted coiling), the aneurysm was treated by a flow diverter. Endovascular treatment was performed under general anesthesia and systemic intravenous heparin. If stent-assisted coiling or flow diverter therapy was chosen, 100 mg/day aspirin and 75 mg/day clopidogrel were administered for at least 5 days before the procedure. Systemic intravenous heparin was administered in patients who underwent an endovascular procedure to maintain an activated clotting time between 250 and 300s, to prevent embolic events. Patients were treated with coiling, stent-assisted coiling, or flow diverter therapy as appropriate. After the procedure, patients treated with the conventional stent were given 75 mg/day clopidogrel for 6 weeks and 100 mg/day aspirin for 6 months, while patients treated with the flow diverter were given 75 mg/day clopidogrel for 3 months and 100 mg/day aspirin thereafter.

### Statistical analysis

Data are presented as the mean ± standard deviation for quantitative variables and frequency for qualitative variables. Risk factors associated with periprocedural ischemic complications were analyzed using an independent sample *t*-test, or a *χ*^2^ test applied as appropriate. Then, variables with p < 0.20 in the univariate logistic analysis were included in the multivariate logistic regression. Statistical significance was considered as *p* < 0.05. Statistical analyses were performed using IBM SPSS Statistics for Windows, v.22.0 (IBM Corp., Armonk, NY, USA).

## Results

### Clinical and aneurysmal characteristics

In total, from August 2016 to March 2017, 167 patients with 167 aneurysms were included in this study. The patients consisted of 107 females and 60 males. The mean age of the patients was 56.17 ± 10.13 years. The mean size of the aneurysms was 8.10 ± 5.50 mm. Of the 167 aneurysms, 37 aneurysms were treated with coiling alone, 93 aneurysms with stent-assisted coiling and 37 aneurysms with flow diverter therapy.

### Periprocedural ischemic stroke

In the current study, endovascular procedures were performed for the 167 patients with 167 aneurysms. Among the 167 patients, periprocedural ischemic stroke occurred in 20 procedures (11.98%). The 20 periprocedural ischemic strokes consisted of 2 intraprocedural thrombus formations, 7 minor ischemic complications, and 11 major ischemic complications. Among the 20 periprocedural ischemic strokes, 7 cases presented with transient neurologic symptoms that resolved on discharge and 13 cases showed persistent neurologic morbidity (7.78%). No mortality was observed.

### Risk factors for periprocedural ischemic stroke

**Fig. 1:**
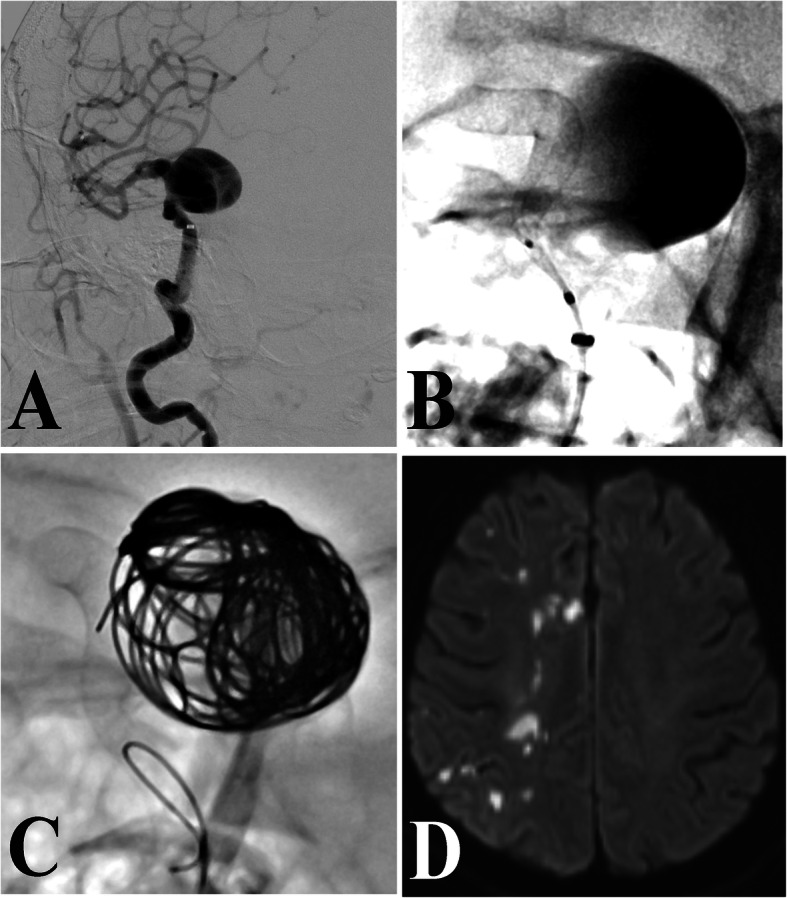
A patient with a large aneurysm was treated by flow diverter with loose packing. **A** Digital subtraction angiogram showed the large aneurysm. **B** The pipeline embolization device was deployed. **C** Loose coils package was formed in the aneurysm sac. Five hours after the endovascular procedure, the patient presented with the weakness of the right limb. **D** Magnetic resonance imaging showed the infarct lesion

As shown in Table 1, after univariate analysis, aneurysm size was significantly correlated with occurrence of periprocedural ischemic stroke. The ischemic group had a higher proportion of large aneurysms (≥ 10 mm) than the control group (45.0% vs. 23.1%, p = 0.036). Moreover, the incidence of periprocedural ischemic stroke was higher in cases treated by flow diverter therapy (21.6%) or stent-assisted coiling (11.8%) than in cases treated by coiling only (2.7%), and the difference was statistically significant (*p* = 0.043) (Fig. 1). The other clinical and aneurysmal factors examined (age, sex, smoking, drinking, hypertension, hyperlipidemia, previous ischemic stroke, shape, location, sidewall/bifurcation and distal/proximal to the circle of Willis) showed no significant differences between the ischemic and control groups (*p*>0.05).

**Table 1:** Results from univariate statistical analysis for all variables

Characteristics	Control group(*N*=147)	Ischemic group (*N*=20)	*P* value
Age (years)	55.8±9.8	58.7±12.4	0.227
Sex (%)			0.556
Male	54 (36.7)	6 (30.0)	
Female	93 (63.3)	14 (70.0)	
Smoking (%)	21 (14.3)	4 (20.0)	0.735
Drinking (%)	20 (13.6)	1 (5.0)	0.466
Hypertension (%)	69 (46.9)	12 (60.0)	0.273
Hyperlipidemia	42 (28.6)	5 (25.0)	0.739
Previous ischemic stroke	23 (15.6)	3 (15.0)	>0.999
Aneurysm size			0.036
<10 mm	113 (76.9)	11 (55.0)	
≥ 10 mm	34 (23.1)	9 (45.0)	
Shape (%)			0.556
Regular	93 (63.3)	14 (70.0)	
Irregular	54 (36.7)	6 (30.0)	
Location (%)			0.479
Anterior circulation	136 (92.5)	17 (85.0)	
Posterior circulation	11 (7.5)	3 (15.0)	
Sidewall/bifurcation aneurysm (%)			0.691
Sidewall	102 (69.4)	13 (65.0)	
Bifurcation	45 (30.6)	7 (35.0)	
Distal aneurysm	42 (28.6)	8 (40.0)	0.295
Treatment therapy (%)			0.043
Coiling	36 (24.5)	1 (5.0)	
Stent-assisted coiling	82 (55.8)	11 (55.0)	
Flow diverter	29 (19.7)	8 (40.0)

### Multivariate logistic regression analysis

The multivariate logistic regression results are presented in Table 2. After multivariate logistic regression analysis, treatment modality was found to be the only independent risk factor for periprocedural ischemic stroke. Compared with the coiling-only procedure, flow diverter therapy was associated with a significantly higher rate of periprocedural ischemic stroke (OR 9.931; 95% CI 1.174–84.038; *p* = 0.035).

**Table 2:** Multivariate logistic regression results

Variables	OR, 95% CI	*p* value
Aneurysm size		
<10 mm		Ref
≥ 10 mm	1.773 (0.587, 5.356)	0.310
Treatment therapy		
Coiling		Ref.
Stent-assisted coiling	4.829 (0.601, 38.822)	0.139
Flow diverter	9.931 (1.174, 84.038)	0.035

## Discussion

Periprocedural ischemic stroke is a major complication of endovascular treatment and is a major cause of neurologic morbidity or even mortality. In the current study, using a real-world database, we comprehensively evaluated potential risk factors associated with periprocedural ischemic stroke after endovascular treatment of intracranial aneurysm. A main finding is that aneurysm size and endovascular treatment modality were associated with the occurrence of periprocedural ischemic stroke. Treatment modality was the only independent risk factor for periprocedural ischemic stroke in this study. We believe the results of this study could provide a beneficial reference for physicians.

Larger aneurysms are associated with increased risk of periprocedural ischemic stroke [12]. Intra-aneurysmal clot is more frequent in larger aneurysms before endovascular treatment, and larger aneurysms are more likely to have residual flow within the coil mass than small aneurysms [13–15]. Furthermore, for stent deployment, large size aneurysms can make it difficult to achieve good wall apposition, thus increasing the risk of periprocedural ischemic stroke [8]. The present study also found that the ischemic group had a higher proportion of large aneurysms than the control group.

Treatment modality was also associated with periprocedural ischemic stroke after endovascular treatment. Compared with coiling treatment alone, the risk of ischemic events is particularly high after stent-assisted coiling or flow diverter deployment, which may be due to the thrombogenicity of intra-arterial devices, longer procedure times, and the complexity of the procedures [16]. Moreover, the disturbed flow across the aneurysm neck with ingress and egress through the stent lumen may also increase the risk of periprocedural ischemic stroke [17]. In this study, treatment modality was the only independent risk factor for periprocedural ischemic stroke.

Periprocedural ischemic stroke mainly results from embolic events during endovascular treatment of intracranial aneurysms, such as the stent wall thrombus, original thrombus, or fresh clot migrating distally during the procedure [18]. Because platelets are the primary component of thrombi, inhibition of platelet reactivity can reduce the occurrence of ischemic complications, especially for stent deployment therapy [14]. Therefore, dual antiplatelet therapy (aspirin and clopidogrel) is widely accepted as the standard protocol to decrease the risk of ischemic complications for intracranial aneurysms treated with stents [17]. However, many patients still suffer periprocedural ischemic stroke after stent deployment, even though the standard antiplatelet medication protocol is applied [18]. Therefore, individualized antiplatelet therapy still needs to be investigated.

There are several limitations of our study. First, a limited number of cases, retrospective design and patient selection bias may limit the generalization of the results. Also, computerized tomography and magnetic resonance are not part of the routine clinical examination after endovascular treatment, so some asymptomatic ischemic complications may not have been detected. Finally, long-term follow-up results and the role of platelet function monitoring were not included in the study; these will be reported in future work.

## Conclusions

Ischemic stroke was the most common procedure-related complication of endovascular treatment. Larger aneurysms increased the risk of periprocedural ischemic stroke. Treatment modality was the independent risk factor of periprocedural ischemic stroke found in this study. Flow diverter therapy resulted in significantly more periprocedural ischemic stroke than the coiling procedure alone.

## Data Availability

In addition to the data published within this article, anonymized data can be obtained by request by any qualified investigator.

## Abbreviations

HARET: Haemodynamic analysis for recanalisation of intracranial aneurysms after endovascular treatment
OR: Odds ratio
CI: Confidence interval
